# A Japanese neonatal case of glucose-6-phosphate dehydrogenase deficiency presenting as severe jaundice and hemolytic anemia without apparent trigger

**DOI:** 10.1186/2193-1801-2-434

**Published:** 2013-09-04

**Authors:** Shinya Tsuzuki, Moe Akahira-Azuma, Masao Kaneshige, Kazuhiro Shoya, Shinichi Hosokawa, Hitoshi Kanno, Takeji Matsushita

**Affiliations:** Department of Pediatrics, National Center for Global Health and Medicine (NCGM), 1-21-1 Toyama, Shinjuku-ku, 162-0855 Tokyo, Japan; Department of Transfusion Medicine and Cell Processing, Tokyo Women’s Medical University, Tokyo, Japan

**Keywords:** Glucose-6-phosphate dehydrogenase deficiency, Neonate, Jaundice, Hemolytic anemia

## Abstract

**Background:**

Glucose-6-phosphate dehydrogenase (G6PD) deficiency is rare among Japanese ethnicity although it is known as one of the most common hereditary disorders of erythrocytes, causing intravascular hemolysis. It is well-known that G6PD deficiency may cause hemolysis even in the neonatal period. However, most cases are asymptomatic, and the frequency of severe anemia is low.

**Findings:**

We describe a Japanese male neonatal case of G6PD deficiency presenting as severe, persistent indirect hyperbilirubinemia on day 2 and hemolytic anemia. He was born to non-consanguineous Japanese parents without any family history. We could not find any triggers that could have induced hemolysis during pregnancy.

**Conclusions:**

This case encouraged us to investigate G6PD deficiency as a differential diagnosis of severe neonatal jaundice and hemolytic anemia despite the low prevalence in Japan.

## Introduction

Glucose-6-phosphate dehydrogenase (G6PD) deficiency is said to be a rare disorder in Japan (Nakashima et al. 1977), even though it is one of the most common genetic enzymatic disorders in the world (Steiner & Gallagher 2007). The characteristic normal course of most G6PD-deficient children includes no symptoms throughout their life, and severe hyperbilirubinemia and kernicterus are seldom observed in the newborn period (Cappellini & Fiorelli 2008). In addition, acute severe hemolysis is thought to occur with the presence of triggering factors, such as infection, drugs, and fava beans (Cappellini & Fiorelli 2008; WHO Working Group 1989; Kaplan et al. 1998).

We describe a neonatal case of G6PD deficiency that developed severe jaundice and acute hemolytic anemia in the absence of ABO incompatibilities. The patient was successfully treated and avoided complications.

## Case report

A male neonate was born at 38+5 weeks gestation to a 40-year-old gravida 2, para 2, blood group O Rh positive mother, via spontaneous vaginal delivery. The pregnancy was uncomplicated. Both his mother and father were non-consanguineous Japanese parents without any past medical history. His sibling (2-year-old girl) did not have any jaundice. His birth weight was 3460 g (appropriate for gestational age), and his Apgar score were 9 at 1 min and 9 at 5 min. He was exclusively breastfed, although the mother’s breast milk secretion was not sufficient.

On day 2, he looked icteric, and a blood test was performed. His serum total bilirubin (STB) was 20.1 mg/dl (the indication of phototherapy according to the criteria of Kobe University, Japan: 12 mg/dl) (Department of Pediatrics, Kobe University Graduate School of Medicine 1993), his serum direct bilirubin was 1.29 mg/dl (normal upper limit: 0.6 mg/dl). He was transferred to our NICU due to severe jaundice and was admitted on the same day.

Physical examinations revealed a body weight of 3217 g (7.0% weight loss). His vital signs were within normal limits. His bulbar conjunctiva and skin were icteric, and no hepatosplenomegaly was found. His blood type was group B, Rh positive. A complete blood count (CBC) revealed hemoglobin (Hb) of 12.6 g/dl and, a reticulocyte count of 10.9%; a blood smear for red cell morphology was within normal limits, without spherocytosis or red cell fragments. Chemistry showed no abnormal findings, except for STB (18.7 mg/dl). Direct and indirect Coombs’ tests were negative. Blood culture was negative. Urinalysis did not show any occult blood, urobilinogen or bacteria. Chest and abdominal radiographs were normal. Head ultrasonography (US) showed no intracranial hemorrhage and abdominal US did not reveal any adrenal hemorrhage.

An exchange transfusion was reserved because of the patient’s STB (the criteria for indication was an STB>20 mg/dl, according to Kobe University), and phototherapy was initiated. Table 1 shows the course of the blood test results and management. A total of 15 ml/kg of red blood cells (RBCs) was transfused on 2 consecutive days (days 16 and days 17). After the RBCs transfusion, a CBC showed 14.3 g/dl of Hb. On day 16, an erythrocyte osmotic fragility test, CD55 and CD59 were within normal limits. He was referred to Tokyo Women’s Medical University to further investigate any erythrocyte disorders. His glucose-6-phosphate dehydrogenase activity was 3.06 IU/gHb (his father and mother’s activity was not available), while the normal level is 11.0 IU/gHb. The patient was diagnosed as having a G6PD deficiency. Two weeks after birth, his jaundice and anemia became stable without any treatment and he was discharged on day 21 with an STB value of 14.7 mg/dl, Hb of 12.9 g/dl and a reticulocyte count of 2.0%.

**Table 1:** **Serum bilirubin, hematological values and other managements during hospitalization**

DOL	2	5	9	13	16	17	31	75	94	149
**Blood Test**										
**Hb (g/dl)**	12.6	12.0	9.4	8.6	7.0	14.3	10.8	8.4	7.9	9.1
**Hct (%)**	37.1	35.6	28.1	25.5	20.6	40.8	31.4	24.1	23.7	28.1
**Ret (%)**	10.9	5.4	2.5	4.1	4.0	2.2	0.5	4.6	7.5	8.6
**STB (mg/dl)**	18.7	18.5	14.4	19.2	14.7	14.6	13.9	4.2	2.0	2.0
**Management**										
**Phototherapy**^**†**^	+	+	-	+	-	-	-	-	-	-
**Transfusion**					*	*				
**Other tests**^**‡**^					**+**			**+**		

Abbreviations: *DOL* Day of life, *Hb* Hemoglobin, *Hct* Hematocrit, *Ret* Reticulocyte, *STB* Serum total bilirubin.

† treatment with phototherapy is indicated with a +.

‡ an osmotic fragility test was performed on DOL 16; an erythrocyte enzyme test was performed on DOL 75.

## Discussion

We treated a Japanese male with severe and refractory jaundice and progressive, early onset normocytic anemia born to non-consanguineous parents. The signs of hemolysis were obscure at the onset of jaundice. Rh, ABO compatibilities and spherocytosis were not observed. Because the erythrocyte enzyme test is not easily accessible in Japan, the infant was referred to a specific institute, and was eventually diagnosed as having a G6PD deficiency on day 75. The instructive aspects of the present report are as follows. First, although G6PD deficiency is uncommon in Japan, the patient was born to Japanese parents. Second, the case presented as severe jaundice and hemolytic anemia, although most cases are asymptomatic, particularly in the neonatal period. Third, he apparently did not have any triggers for an acute episode of hemolysis.

The prevalence of G6PD deficiency is estimated at approximately 0.1% in Japan (Nakashima et al. 1977). In most hemolytic cases, it was inferred that the mothers were of non-Japanese ethnicity because gene variants found in Japan were not associated with severe hemolytic anemia (Nakashima et al. 1977). This also might be attributed to the fact that the incidence of gene mutation was quite different from southern Chinese in the Taiwan-Hakka population and Philippines (5.5% and 6.0% each) (Nakashima et al. 1977). In fact, case reports of G6PD deficiency with severe hemolytic anemia published in Japan often describe the family history of foreign mothers (Shimomura et al. 2002; Akazawa et al. 2011). Therefore this case was also characteristic in the point that both the father and mother are Japanese.

Most G6PD-deficient individuals are entirely asymptomatic (Mehta 1994). The most common clinical manifestation in the neonatal period is neonatal jaundice, and acute hemolytic anemia is usually rare; this is because neonatal jaundice due to G6PD deficiency is not due to hemolysis, and hyperbilirubinemia is thought to be secondary to reduced hepatic conjugation and bilirubin excretion (Cappellini & Fiorelli 2008; Beutler 1994; Kaplan & Hammerman 2002). Our case presented neonatal jaundice in advance of the hemolytic anemia; however, the increased reticulocyte count on day 2 suggests that both hyperbilirubinemia and hemolysis may occur simultaneously. Kawaguchi et al. also reported a suspected antenatal hemolysis case in Japan (Kawaguchi et al. 1997). In this case, an extremely high reticulocyte level (344‰) was presented, and intrauterine hemorrhage was strongly suspected. They attributed the early onset hemolysis to oxidative stress as a result of maternal history of the common cold or drug use.

This illness generally manifests as acute hemolysis in childhood, which usually arises when red blood cells undergo oxidative stress triggered by infection or the ingestion of fava beans. Despite conducting a thorough and precise interview, we could not identify any history of drug or food intake during the pregnancy. There have been a few G6PD deficiency cases presenting acute hemolysis in the absence of any trigger that have also been reported in the literature (Dhillon et al. 2003; Shah & Yeo 2007). Our case supports the theory that massive hemolysis may occur in neonates with G6PD deficiency even in the absence of obvious triggering factors.

The abnormal genetic mutations of this disease are classified into 3 categories. Class V/IV is clinically asymptomatic, and class III/II (the border between III and II is not obvious) is basically asymptomatic; both are without the risk of hemolytic anemia and neonatal jaundice. Class I presents (severe) neonatal jaundice and acute exacerbation of hemolytic anemia (WHO Working Group 1989). The limitation of our report is that this case should be classified as class I because the patient showed both acute and chronic hemolytic episodes. However, no genetic testing has been performed. Differences in the clinical course due to genetic transmutation may be present in this disorder. Therefore, genetic testing is one of important part of the management of G6PD patients.

The Japanese male neonate with G6PD deficiency in this report simultaneously presented with severe jaundice and acute hemolysis. Given the lack of family history, triggers, definite laboratory data, and rare disease status among patients with Japanese ethnicity, we recommend further investigation for G6PD deficiency in cases of neonatal hemolytic anemia complicated with jaundice.

## Abbreviations

G6PD: Glucose-6-phosphate dehydrogenase
STB: Serum total bilirubin
CBC: Complete blood count
Hb: Hemoglobin
US: Ultrasonography
RBCs: Red blood cells.
